# Dr. Wm. H. Annowski

**Published:** 1914-09

**Authors:** 


Dr. Wm. H. Annowski, Buffalo, 1893, died suddenly of apoplexy on his yacht, off Crystal Beach, Ont., August 1, aged 63. He was born and had lived all his life in Buffalo, serving 35 years in the Police Dept., as Desk Sergeant, up to June 30, 1913, when he was retired from the service.

